# Differential Regulation of Self-reactive CD4^+^ T Cells in Cervical Lymph Nodes and Central Nervous System during Viral Encephalomyelitis

**DOI:** 10.3389/fimmu.2016.00370

**Published:** 2016-09-21

**Authors:** Carine Savarin, Cornelia C. Bergmann, David R. Hinton, Stephen A. Stohlman

**Affiliations:** ^1^Department of Neurosciences, NC-30, Lerner Research Institute, Cleveland Clinic Foundation, Cleveland, OH, USA; ^2^Department of Pathology, Keck School of Medicine, University of Southern California, Los Angeles, CA, USA

**Keywords:** autoimmunity, central nervous system, viral infection, CD4^+^ T cells, Foxp3^+^ regulatory T cells, Tr1 cells

## Abstract

Viral infections have long been implicated as triggers of autoimmune diseases, including multiple sclerosis (MS), a central nervous system (CNS) inflammatory demyelinating disorder. Epitope spreading, molecular mimicry, cryptic antigen, and bystander activation have been implicated as mechanisms responsible for activating self-reactive (SR) immune cells, ultimately leading to organ-specific autoimmune disease. Taking advantage of coronavirus JHM strain of mouse hepatitis virus (JHMV)-induced demyelination, this study demonstrates that the host also mounts counteractive measures to specifically limit expansion of endogenous SR T cells. In this model, immune-mediated demyelination is associated with induction of SR T cells after viral control. However, their decline during persisting infection, despite ongoing demyelination, suggests an active control mechanism. Antigen-specific IL-10-secreting CD4^+^ T cells (Tr1) and Foxp3^+^ regulatory T cells (Tregs), both known to control autoimmunity and induced following JHMV infection, were assessed for their relative *in vivo* suppressive function of SR T cells. Ablation of Foxp3^+^ Tregs in chronically infected DEREG mice significantly increased SR CD4^+^ T cells within cervical lymph nodes (CLN), albeit without affecting their numbers or activation within the CNS compared to controls. In contrast, infected IL-27 receptor deficient (IL-27R^−/−^) mice, characterized by a drastic reduction of Tr1 cells, revealed that SR CD4^+^ T cells in CLN remained unchanged but were specifically increased within the CNS. These results suggest that distinct Treg subsets limit SR T cells in the draining lymph nodes and CNS to maximize suppression of SR T-cell-mediated autoimmune pathology. The JHMV model is thus valuable to decipher tissue-specific mechanisms preventing autoimmunity.

## Introduction

Viruses have long been associated with autoimmune diseases of the central nervous system (CNS), such as multiple sclerosis (MS), both as potential inducers and as facilitators of a self-reactive (SR) immune response (1, 2). While the detection of viral antigens and antiviral antibodies in MS brains supports a role for viral infections in triggering autoimmunity (3–5), the “hygiene hypothesis” rather proposes a protective function (6, 7), underlining the ambiguous link between viral infections and MS (8). This dichotomy between viral infections and autoimmune diseases is reflected in two prominent models of viral-induced demyelination, i.e., Theiler’s murine encephalomyelitis virus (TMEV) and neurotropic mouse hepatitis virus (MHV). During persistent TMEV infection, activation of SR T cells by epitope spreading triggers an autoimmune disease characterized by demyelination and ascending paralysis (9, 10). By contrast, despite ongoing demyelination, persistent neurotropic MHV infections are defined by clinical recovery with no evidence for autoimmune-mediated clinical symptoms (11, 12). Nevertheless, our recent study demonstrated that endogenous SR T cells are activated specifically during chronic infection, correlating with kinetics of JHM strain of mouse hepatitis virus (JHMV)-induced demyelination (13). However, the endogenous SR T cell response declined despite sustained myelin loss during viral persistence and the preserved ability of antigen-presenting cells from both cervical lymph nodes (CLN) and the CNS to activate myelin-specific CD4^+^ T cells *ex vivo*. These findings suggested that chronic JHMV infection actively induces suppressive mechanisms to limit the development of autoimmunity.

Immune regulation associated with viral infections requires a delicate balance between promoting effective virus clearance while limiting tissue damage. This is especially critical within the CNS due to its limited regenerative capacity. Following JHMV infection, T cells primarily control infectious virus within the brain and spinal cords within 2 weeks. Nevertheless, viral persistence is evident by ongoing detection of viral RNA predominantly within spinal cords. Demyelination is initiated during T cell-mediated control of viral replication, peaks subsequent to virus control, but continues throughout persistence (12). Induction of Foxp3^+^ regulatory T cell (Tregs) and type 1 regulatory T (Tr1) cells and their migration to the CNS are already evident during the acute infection (14, 15). Both regulatory populations are known to control autoimmune diseases (16, 17). Prior studies of their role during JHMV infection demonstrated that Tr1 cells have a limited effect on tissue damage (15), while Foxp3^+^ Tregs restrict the extent of demyelination without affecting viral persistence (18, 19). Indeed, depletion of CD25^+^ regulatory CD4^+^ T cells prior JHMV infection correlated with increased myelin loss during viral persistence (19). Similarly, transfer of naive Foxp3^+^ Tregs into acutely JHMV-infected mice improved clinical disease and restricted tissue damage without altering control of virus replication (20). The limited role of Foxp3^+^ Tregs in virus clearance was also confirmed by unaltered antiviral T cell responses following Foxp3^+^ Treg depletion during acute infection with the less pathogenic MHV-A59 strain (21). However, the same study showed that Foxp3^+^ Tregs limited proliferation of transferred SR T cells in the CLN (21). Altogether, these data support the notion that Foxp3^+^ Tregs limit tissue damage by specifically counteracting the activation of endogenous SR T cells, without apparent affects on antiviral T cells. However, in all these studies, transfer or depletion of Foxp3^+^ Tregs were performed during acute MHV infection, prior to the induction of endogenous SR T cells (13), limiting knowledge of their function during persistence. Regulation of *in vivo* primed SR T cells and immunopathology by endogenous Foxp3^+^ Treg during persistence remains unexplored.

To assess how Foxp3^+^ Treg depletion affects endogenous SR T cells during JHMV persistence, we chose DEREG mice, which express the human diphtheria toxin (DT) receptor under the control of the Foxp3 promoter (22). Incomplete DT-mediated Foxp3^+^ Treg depletion in naive adult DEREG mice is advantageous to our studies as it enables a window to study effects of Foxp3^+^ Treg ablation on myelin reactive CD4^+^ T cells without confounding complications of lymphoproliferative disease and systemic lethal autoimmunity (23, 24). DT treatment of JHMV-infected DEREG mice at the peak of SR T cell CNS infiltration (between days 21 and 28 post infection), resulted in increased lymphocyte expansion and T cell activation in CLN, coincident with elevated pro-inflammatory cytokine expression compared to DT-treated controls. More importantly, Foxp3^+^ Treg ablation specifically increased frequencies of myelin-specific but not virus-specific CD4^+^ T cells, indicating preferential regulation of peripheral SR T cells by Foxp3^+^ Tregs. Surprisingly however, CNS inflammation, viral persistence, and demyelination remained similar consistent with no changes in virus and myelin-specific CD4^+^ T cells within the CNS. The apparent redundant role of Foxp3^+^ Tregs in regulating CNS inflammation implied a potential protective function of Tr1 cells. Analysis of SR T cells during chronic JHMV infection of IL-27R^−/−^ mice, which lack Tr1 cells, revealed no effects within the CLN. By contrast, both virus-specific and SR CD4^+^ T cells were increased within the CNS of IL-27R^−/−^ relative to wild-type (WT) mice. Altogether, these data indicate differential regulation of SR CD4^+^ T cells within the CLN versus CNS during chronic JHMV infection. While Foxp3^+^ Tregs specifically control myelin-specific CD4^+^ T cells within CLN, Tr1 cells limit SR T cells within the CNS.

## Materials and Methods

### Mice, Infection, and Foxp3^+^ Treg Depletion

C57BL/6 WT mice were purchased from the National Cancer Institute (Frederick, MD, USA). C57BL/6 DEREG mice, which express the enhanced green fluorescent protein (eGFP) and diphtheria toxin receptor (DTR) under the control of the Foxp3 promoter, were kindly provided by Dr. T. Sparwasser (Twincore, Hannover, Germany) (22). C57BL/6 homozygous IL-27Rα (WSX-1) deficient (IL-27R^−/−^) mice were provided by Dr. C. Saris (Amgen, Thousand Oaks, CA, USA). Mice were bred and maintained at the Biological Resources Unit of the Cleveland Clinic Lerner Research Institute under sterile conditions. All procedures were performed in compliance with protocols approved by the Cleveland Clinic Institutional Animal Care and Use Committee. Mice of both sexes at 6–7 weeks of age were infected in the left hemisphere with 1,000 plaque forming unit (PFU) of the sublethal gliatropic JHMV monoclonal antibody (mAb) derived variant designated 2.2v-1 (25) in 30-μl endotoxin-free Dulbecco’s phosphate-buffered saline (PBS). Mice were assessed daily for clinical disease severity according to the following scale: 0, healthy; 1, hunched back and ruffled fur; 2, partial hind limb paralysis or inability to maintain the upright position; 3, complete hind limb paralysis; 4, moribund or dead. For Foxp3^+^ Treg depletion, DEREG mice and control littermates (Foxp3^eGFP^DTR^−^ mice) received daily intraperitoneal (i.p.) injections of 1-μg DT (EMD Biosciences, San Diego, CA, USA) between days 21 and 28 post infection (p.i.).

### Tissue Processing for Cell Isolation

Mice were perfused with ice cold PBS before harvesting CLN, spleen (SPL), brain, and spinal cord. CLN were dissociated through a cell strainer and single cell suspensions washed and resuspended in RPMI 1640 medium. For splenocyte isolation, tissues were dissociated, treated with Gey’s solution to lyse red blood cells, and washed twice in RPMI 1640 medium. To isolate CNS infiltrating cells, brains and spinal cords were homogenized in 4-ml Dulbecco’s PBS using TenBroeck tissue grinders as described (26). Tissue homogenates were adjusted to 30% Percoll (Pharmacia, Uppsala, Sweden) and underlayed with 1-ml 70% Percoll. Following centrifugation at 850 *g* for 30 min at 4°C, mononuclear cells were recovered from the 30–70% interface and washed with RPMI 1640 medium.

### Flow Cytometry

Cells were resuspended in FACS buffer (Dulbecco’s PBS + 1% bovine serum albumin). Non-specific staining was blocked with mouse serum and anti-mouse CD16/CD32 (clone 2.4G2, BD Biosciences, San Jose, CA, USA) for 15 min on ice. Cells were stained for 30 min on ice with fluorescein isothiocyanate (FITC), phycoerythrin (PE), peridin chlorophyll protein (PerCP), or allophycocyamin (APC) conjugated mAb (all for BD Biosciences expect when indicated) specific for CD45 (clone Ly-5), CD4 (clone GK1.5), CD8 (clone 53-6.7), CD11b (M1/70), CD25 (clone PC61), MHC class II (clone 2G9), CD44 (clone IM7), CD62L (clone MEL-14), CD69 (clone H1-2F3), and CXCR3 (R&D Systems, Minneapolis, MN, USA). Virus-specific CD8 T cells were characterized using H-2D^b^/S510 MHC class I tetramers (27). Intracellular staining for Foxp3 (eBioscience, San Diego, CA, USA) was performed according to the manufacturer’s instructions. Cells were washed twice with FACS buffer and analyzed using a FACSCalibur flow cytometer (BD Biosciences) and FlowJo software (Tree Star Inc., Ashland, OR, USA).

### RNA Extraction, Reverse Transcription, and Gene Expression Analysis

Snap-frozen tissues were homogenized in TRIzol (Invitrogen, Carlsbad, CA, USA) using a TissueLyser and stainless steel beads (Qiagen, Valencia, CA, USA). RNA was extracted according to manufacturer’s instructions. Briefly, RNA was extracted by adding chloroform prior to centrifugation at 12,000 *g* for 15 min at 4°C. RNA was then precipitated with isopropyl alcohol, washed with 75% ethanol, and resuspended in RNase-free water. DNA contamination was eliminated by DNase I treatment for 30 min at 37°C (DNA-free kit, Ambion, Austin, TX, USA). cDNA was synthesized by reverse transcription using Moloney murine leukemia virus reverse transcriptase (Invitrogen) and a mixture of oligo(dT) (10 mM) (Promega, Madison, WI, USA) and 250 ng random primers (Promega). Gene expression analysis was performed by quantitative real-time PCR (qPCR) using SYBR Green or Taqman master mixes on a 7500 Fast real-time PCR system (Applied Biosystems, Foster City, CA, USA). SYBR Green primers used for transcripts were as follows: GADPH, 5′-TGCACCACCAACTGCTTAG-3′ (sense) and 5′-GGATGCAGGGATGATGTTC-3′ (antisense); JHMV nucleocapsid, 5′-CGCAGAGTATGGCGACGAT-3′ (sense) and 5′-GAGGTCCTAGTCTCGGCCTGTT-3′ (antisense); IL-2, 5′-CAGGATGGAGAATTACAGGAACCT-3′ (sense) and 5′-TTTCAATTCTGTGGCCTGCTT-3′ (antisense); CCL5, 5′-GCAAGTGCTCCAATCTTGCA-3′ (sense) and 5′-CTTCTCTGGGTTGGCACACA-3′ (antisense); CXCL9, 5′-TGCACGATGCTCCTGCA-3′ (sense) and 5′-AGGTCTTTGAGGGATTTGTAGTGG-3′ (antisense); and CXCL10, 5′-GACGGTCCGCTGCAACTG-3′ (sense) and 5′-GCTTCCCTATGGCCCTCATT-3′ (antisense). GAPDH, interferon gamma (IFN-γ), and CCL2 mRNA levels were determined using TaqMan primers. Transcript levels were normalized to the levels of the housekeeping gene GAPDH by using the formula 2[Ct^(GAPDH)^ − Ct^(gene)^] × 1,000, where Ct represents the threshold cycle value.

### Peptides and Enzyme-Linked ImmunoSPOT Assay

Virus (M133)-, myelin oligodendrocyte glycoprotein (MOG)- and myelin basic protein (MBP)-specific CD4 T cells were detected by Enzyme-Linked ImmunoSPOT (ELISPOT) assay as described (13). Briefly, 96-well plates were coated with IFN-γ capture Ab (BD Biosciences). Serial dilutions of cells were cultured for 36 h at 37°C in RPMI complete (RPMI 1640 medium containing 2-mM l-glutamine, non-essential amino acid, 1-mM sodium pyruvate, 25-μg/ml gentamycine, and 5 × 10^−5^M 2-mercaptoethanol) supplemented with 10% fetal calf serum in the presence of stimulator splenocytes preincubated with or without 1-μM peptide (M^133−147^, GTVYVRPIIEDYHT; MOG^35−55^, MEVGWYRSPFSRVVHLYRNGK; MBP^60−80^, SHHAARTTHYGSLPQKSQR; Bio-Synthesis, Lewisville, TX, USA). Spots were detected by sequential incubation with biotinylated anti-IFN-γ mAb (BD Biosciences) overnight at 4°C, horseradish peroxidase conjugated streptavidin (BD Biosciences) for 1 h at room temperature and 3,3′-diaminobenzidine substrate (Sigma–Aldrich, St. Louis, MO, USA). Numbers of spots were determined using a CTL ImmunoSpot analyzer (Cellular Technology Ltd., Shaker Heights, OH, USA). Spots detected in wells with no peptide (negative control) were subtracted from spots formed in the presence of peptide and results presented as the number of IFN-γ secreting cells normalized to 10^6^ input cells.

### Histopathological Analysis

After PBS perfusion, spinal cords were fixed in 10% zinc formalin, divided into six sections (two per cervical, thoracic, and lumbar regions) prior embedding in paraffin. Then, 6 μm cross sections were stained with Luxol fast blue (LFB) to visualize demyelinated lesions. To determine the percentage of demyelination, LFB-stained sections of all six levels per individual mouse were scanned with an Aperio ScanScope (Vista, CA, USA) at 40× and areas of demyelination within the white matter tracks were quantify using Aperio software.

### Statistical Analysis

Data represent the mean ± SEM and were plotted using Graphpad prism 5.0 software (Graphpad Software Inc., La Jolla, CA, USA). Statistical analysis was performed using a Mann–Whitney test, Dunn’s multiple comparison test, and ANOVA with Bonferroni post-test. *p*-Values <0.05 were considered statistically significant.

## Results

### Dynamics of Foxp3^+^ Tregs and Depletion in DEREG Mice during Chronic JHMV Infection

**Figure 1:**
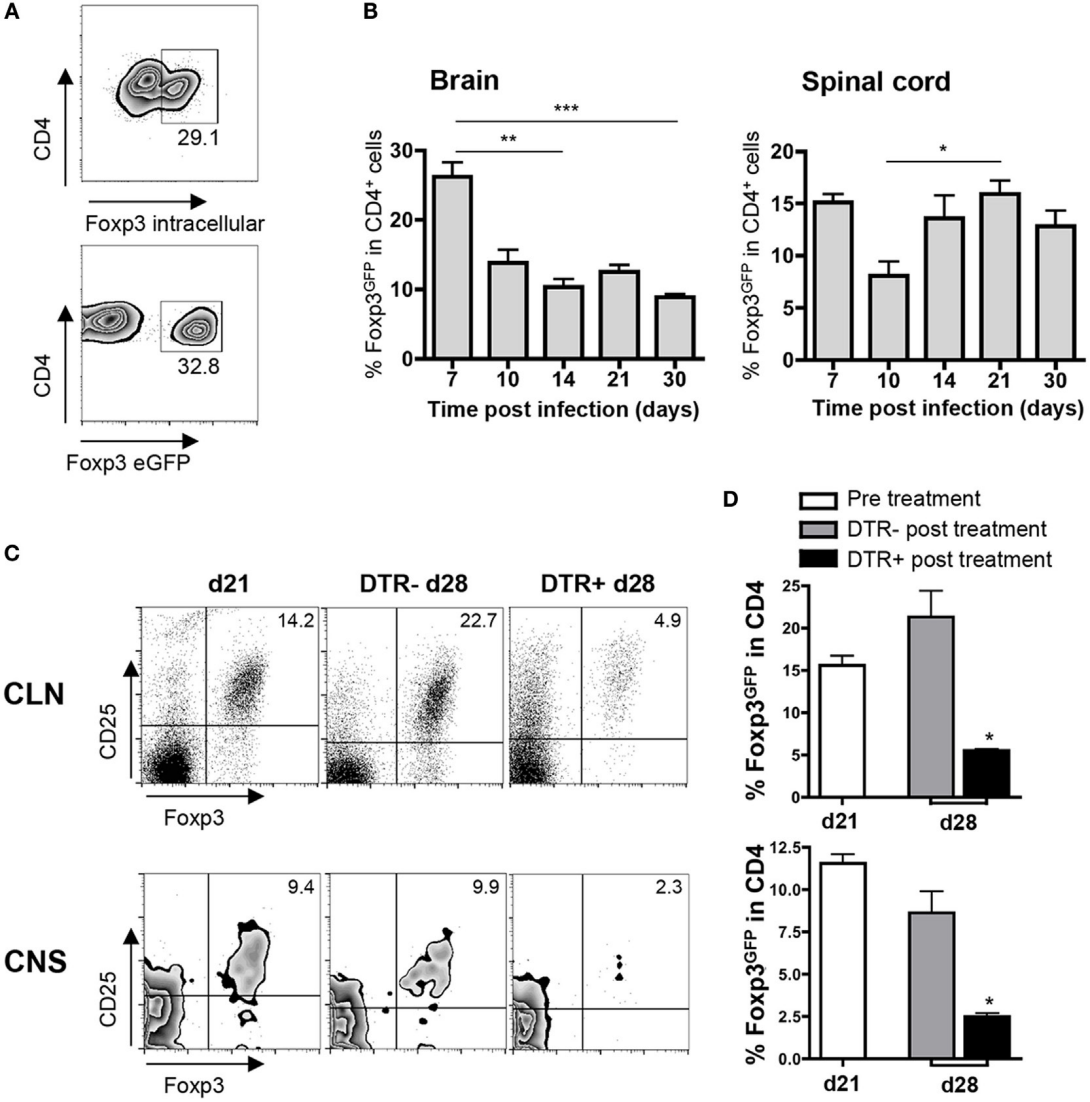
**Kinetics of CNS localized Foxp3^+^ Tregs and depletion in infected DT-treated DEREG mice**. **(A)** Representative flow cytometry density plots depicting intracellular Foxp3 and eGFP staining in brain-derived CD4^+^ T cells from WT and DEREG mice, respectively, at day 7 p.i. **(B)** Frequencies of Foxp3^+^ cells in CD4^+^ T cells derived from brain and spinal cord of infected DEREG mice between days 7 and 30 p.i. Data represent the mean ± SEM of three separate experiments with *n* = 3 per time point per experiment. *p < 0.05, **p < 0.01, ***p < 0.001, Dunn’s multiple comparison test **(C)** Representative flow cytometry plots depicting Foxp3^+^CD25^+^ cells gated on CD4^+^ T cells from CLN and CNS before (d21 p.i.) and after (d28 p.i.) DT treatment of DTR^−^ and DTR^+^ mice. **(D)** Percentage of Foxp3^+^ cells in CD4^+^ T cells from CLN (top) and CNS (bottom) before (d21) and after (d28) DT treatment in DTR^−^ and DTR^+^ mice. Data represent the average ± SEM of four individual experiments. *p < 0.05, Mann–Whitney test.

Foxp3^+^ Tregs infiltrate the CNS during acute JHMV infection and are retained together with both virus-specific and SR T cells during persistence (13, 19). However, their role in regulating endogenously primed T cell populations of viral, and more importantly, SR specificity has not been addressed. To specifically investigate a function of Foxp3^+^ Tregs during JHMV persistence, we infected DEREG mice, in which DT treatment abrogated the vast majority of Foxp3^+^ Tregs under homeostatic conditions but retained a minor fraction thereby circumventing lymphoproliferative disease (22). We first confirmed that transgene expression in DEREG mice does not alter JHMV pathogenesis compared to syngeneic WT mice. Clinical disease onset and progression, viral clearance, and CNS inflammatory responses were similar between the two groups (data not shown). The extent and severity of demyelination within spinal cords was also not altered (data not shown). Moreover, high fluorescent intensity of eGFP expressed under control of the Foxp3 promoter in DEREG mice enabled a distinct separation of the Foxp3^+^ population among CD4^+^ T cells compared to direct intracellular Foxp3-staining in WT mice (Figure 1A). Most importantly, no significant differences in the frequency of Foxp3^+^ cells within CD4^+^ T cells during JHMV infection were found comparing the two groups (data not shown). Validation of Foxp3 expression in DEREG mice thus facilitated characterization of Foxp3^+^ Treg kinetics during the course of infection. The frequency of Foxp3^+^ cells within CD4^+^ cells remained stable at ~15% within CLN at all time points (data not shown). By contrast, within both brain and spinal cord, Foxp3^+^ cells within CD4^+^ T cells peaked at day 7 p.i. and then rapidly decreased at day 10 p.i. (Figure 1B). However, while the frequency of Foxp3^+^ Tregs was maintained at ~10% within the brain after day 10 p.i. (Figure 1B), a sustained rebound was observed in spinal cords after day 14 p.i. during chronic infection (Figure 1B). The sustained increase in the fraction of Foxp3^+^ Tregs at later time points, specifically within the spinal cord, was not attributed to viral replication, as infectious virus is cleared by day 14 p.i., and viral RNA levels continuously decline after day 10 p.i. (15). Onset of robust demyelination in spinal cords at day 14 p.i. (12) rather implicates that the second wave of Tregs is pathology-driven and functions to limit emerging SR CD4^+^ T cell responses.

Based on the delayed expansion of endogenous SR CD4^+^ T cells coincident with onset of demyelination and rebound of Tregs in spinal cords during chronic infection (13), we assessed the effect of Treg depletion during viral persistence. JHMV-infected DEREG mice (DTR^+^) and their control littermates (DTR^−^) were treated daily with DT at the peak of SR CD4^+^ T cell response between days 21 and 28 p.i. (13). Foxp3^+^ Treg frequencies were 70% reduced at day 28 p.i. in both CLN and CNS of DTR^+^ mice (Figures 1C,D). By contrast, control DTR^−^ treated mice revealed no alteration of Foxp3^+^ T cell frequencies in CLN and CNS after DT treatment (Figures 1C,D). These results confirmed the specificity of DT-mediated Foxp3^+^ Treg depletion as well as their residual presence due to incomplete abrogation and rebound after ongoing DT treatment (23, 28).

### Foxp3^+^ Tregs Regulate CLN Inflammation

**Figure 2:**
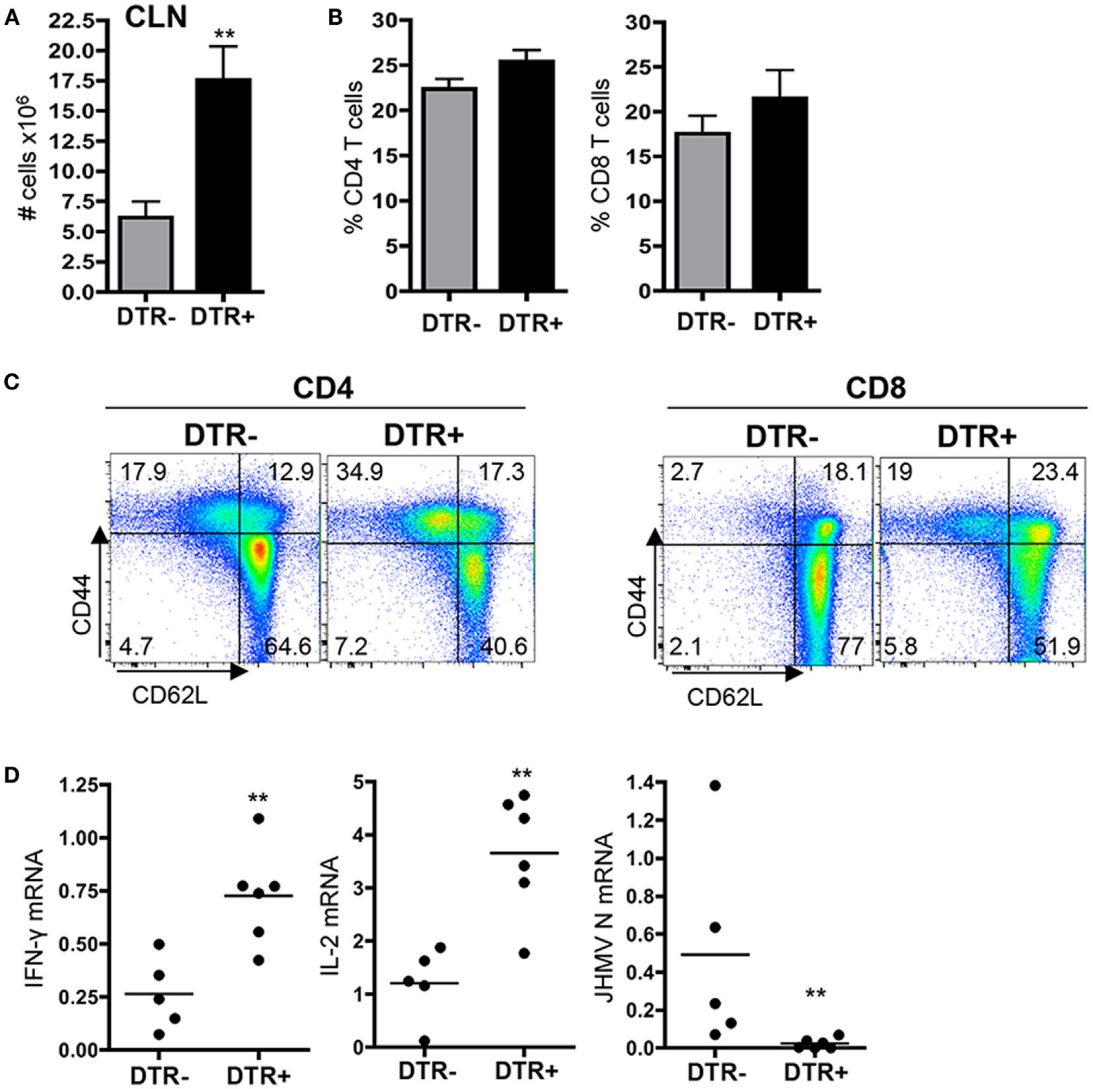
**Increased CLN inflammatory response in absence of Foxp3^+^ Tregs**. **(A)** Total number of cells within CLN of DT-treated DTR^−^ and DTR^+^ mice at d28 p.i. Data represent the mean ± SEM of five to eight individual experiments. **p < 0.01, Mann–Whitney test. **(B)** Frequency of CD4 (*n* = 13) and CD8 (*n* = 4) T cells within CLN of DTR^−^ and DTR^+^ mice treated with DT between days 21 and 28 p.i. Data represent the mean ± SEM **(C)** Flow cytometry dot plots of CD62L and CD44 expression on CD4^+^Foxp3^−^ cells and CD8^+^ T cells derived from CLN of DT-treated DTR^−^ and DTR^+^ mice at d28 p.i., representative of two separate experiments with *n* = 4 per group per experiment. **(D)** IFN-γ, IL-2, and JHMV N mRNA expression relative to GAPDH mRNA within CLN of DT-treated DTR^−^ and DTR^+^ mice at d28 p.i. Dots represent individual mice with *n* = 5–6 per group, and horizontal bars indicate mean number. **p < 0.01, Mann–Whitney test.

Initial T cell activation of both virus- and myelin-specific CD4^+^ T cells occurs within CLN (13, 29), consistent with anatomical linkage between deep CLN and CNS by the meningeal lymphatic system (30). Transfer of Foxp3^+^ Tregs during acute JHMV infection limited CNS tissue damage by inhibiting T cell proliferation within CLN and reducing overall T cell numbers within the CNS, yet without affecting frequencies of virus-specific T cells (20). These studies predicted that Foxp3^+^ Treg depletion during chronic JHMV infection would increase T cell numbers in CLN and CNS. DT-treated DTR^+^ mice indeed exhibited a threefold increase in CLN cellularity compared to DTR^−^ controls between days 21 and 28 p.i. (Figure 2A). However, overall CD4^+^ and CD8^+^ T cell proportions were not altered suggesting the cellular increase was not lymphocyte specific (Figure 2B). Analysis of T cell activation following Treg depletion revealed a significant increase in CD62L^low^CD44^hi^ populations in both the CD4^+^ and CD8^+^ T cell subset (Figure 2C). Contrasting increased activation in CLN, DTR^−^ and DTR^+^ mice showed no difference in total numbers of splenocytes 1 week after DT treatment (data not shown), suggesting specific regulation in CLN.

Enhanced activation specifically in CLN of DT-treated DEREG mice during persistence may be polyclonal and/or driven by low levels of residual viral or auto-antigen presentation in CLN (12, 13). The increased T cell effector cell phenotype indeed correlated with elevated levels of mRNA for T cell derived pro-inflammatory cytokines IFN-γ and IL-2 (Figure 2D). Although there is no evidence suggesting peripheral virus replication during persistent infection, the presence of JHMV within CNS-draining lymph nodes was indeed supported by detection of low levels of mRNA encoding the viral nucleocapsid (N) protein in control DTR^−^ mice (Figure 2D). In contrast, increased T cell activation correlated with a reduction of viral N mRNA to below detection within CLN of DTR^+^ mice at day 28 p.i. (Figure 2D). Since IFN-γ has antiviral activity (31, 32), elevated IFN-γ as a result of increased antiviral T cell activity in the absence of Treg is consistent with a decline in viral RNA.

**Figure 3:**
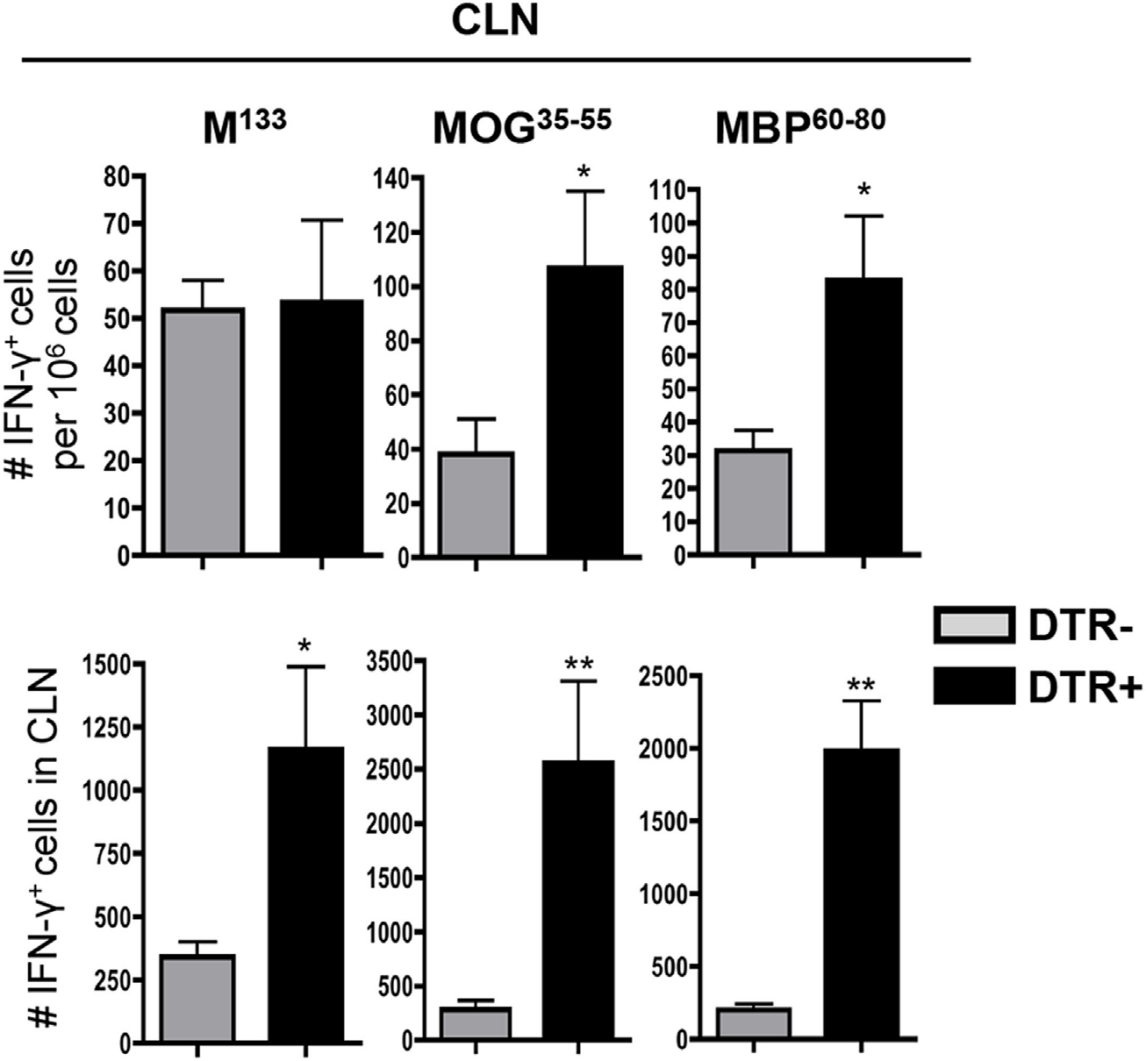
**Foxp3^+^ Tregs control peripheral SR CD4^+^ T cell response during chronic JHMV infection**. Frequencies and absolute numbers of virus-specific M^133^, myelin-specific MOG^35−55^, and MBP^60−80^ CD4^+^ T cells in CLN of JHMV-infected DTR^−^ and DTR^+^ mice treated with DT between d21 and d28 p.i. determined by ELISPOT. The Number of spots detected in wells without stimulation was subtracted to exclude non-specific CD4^+^ T cells. Data represent the mean of two separate experiments with seven to eight pooled mice per group per experiment. *p < 0.05, *p < 0.01, Mann–Whitney test.

To directly assess whether elevated T cell activation is antigen driven or more polyclonal in nature, we measured the frequencies of both virus-specific as well as SR CD4^+^ T cells in CLN following Foxp3^+^ Treg depletion. Frequencies of virus-specific CD4^+^ T cells, as analyzed by IFN-γ secreting cells following viral peptide M^133^ stimulation, were not altered in DTR^+^ compared to DTR^−^ mice (Figure 3). However, reflecting overall increased cellularity, their absolute numbers increased more than threefold (Figure 3). We next assessed how Foxp3^+^ Tregs regulate SR-specific CD4^+^ T cells in CLN. Similar to virus-specific CD4^+^ T cells, myelin-specific endogenous CD4^+^ T cells are activated in CLN, albeit at later times correlating with demyelination (13). More importantly, APC presenting myelin Ag are present in CLN of chronically infected mice (13). Analysis of SR IFN-γ-secreting MOG^35−55^ and MBP^60−80^ specific CD4^+^ T cell frequencies revealed a threefold increase following Foxp3^+^ Treg depletion compared to the very low frequencies in DT-treated DTR^−^ mice (~30 cells per 10^6^ CLN cells; Figure 3). Higher frequencies of SR T cells thus correlated with an overall ninefold increase of MOG^35−55^ and MBP^60−80^ specific CD4^+^ T cell numbers within the CLN of DTR^+^ mice compared to controls (Figure 3). These data indicated that Foxp3^+^ Tregs unspecifically regulate overall CLN cellularity and T cell activation during JHMV persistence but specifically limit expansion of SR CD4 T cells emerging after onset of demyelination.

### Foxp3^+^ Tregs Do Not Regulate CNS Inflammation or Virus Control during Persistence

**Figure 4:**
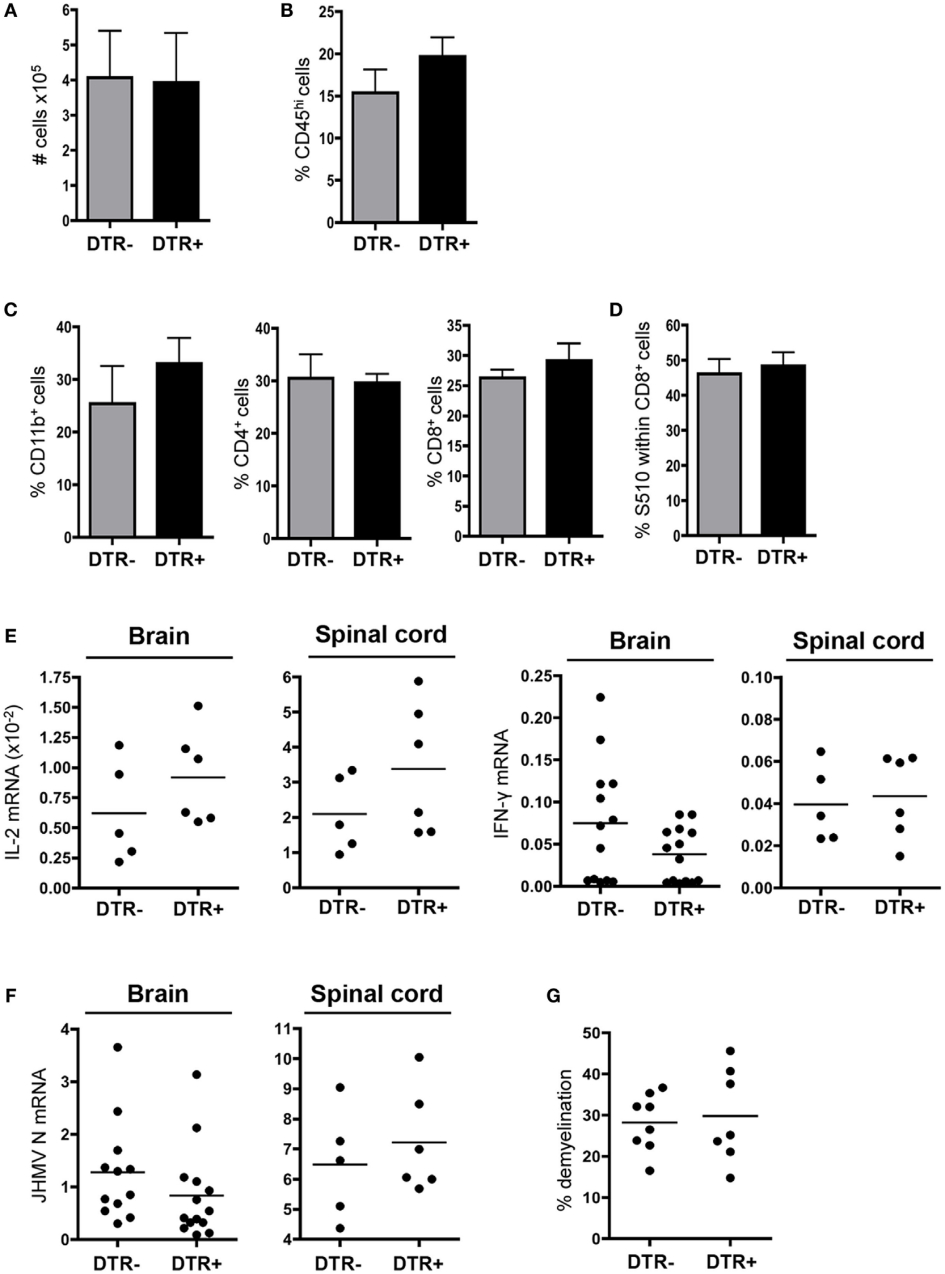
**Foxp3^+^ Tregs do not affect CNS inflammatory response during chronic JHMV infection**. **(A)** Total cell number and **(B)** frequency of CNS infiltrated leukocytes (CD45^hi^) from DT-treated DTR^−^ and DTR^+^ mice at d28 p.i. **(C)** Percentages of CD11b^+^, CD4^+^, and CD8^+^ T cells within CD45^hi^ cells and **(D)** frequencies of virus-specific CD8^+^ T cells analyzed at day 28 p.i. by flow cytometry using H-2D^b^/S510 MHC class I tetramers in DT-treated DTR^−^ and DTR^+^ mice. Data represent the mean ± SEM of at least three individual experiments with *n* = 4 per group per experiment. Levels of mRNA encoding IFN-γ, IL-2 **(E)**, as well as JHMV N **(F)** in brain and spinal cord of DT-treated DTR^−^ and DTR^+^ mice at d28 p.i. analyzed by real-time PCR and relative to GAPDH mRNA expression. Dots represent individual mice with *n* = 5–14, and horizontal bars indicate mean number. **(G)** Percentage of demyelination within white matter area analyzed in the spinal cord of DT-treated DTR^−^ and DTR^+^ mice at d28 p.i. Dots represent individual mouse with *n* = 7–8 mice per group, and horizontal bar indicates mean number.

Increased CLN activation in absence of Foxp3^+^ Tregs suggested CNS inflammatory responses are also increased in analogy to Treg supplementation results by Trandem et al. (20). Surprisingly however, Foxp3^+^ Treg depletion altered neither total CNS cellular infiltration (Figure 4A) nor frequencies of inflammatory leukocytes (Figure 4B) at d28 p.i. Percentages of CD11b^+^, CD4^+^, and CD8^+^ T cells, including virus-specific CD8^+^, all remained similar irrespective of Foxp3^+^ Treg depletion (Figures 4C,D). T cell activation in the CNS cannot readily be deduced by their phenotype as the majority of CD4^+^ and CD8^+^ T cells express a CD44^+^CD62L^low^CD69^+^ effector phenotype throughout infection (27). Nevertheless, as anticipated, we found no phenotypic T cell differences in DT-treated DTR^−^ compared to DTR^+^ mice (data not shown). Similar overall T cell effector function was supported by identical levels of mRNA encoding pro-inflammatory cytokines IFN-γ and IL-2 in both brain and spinal cord (Figure 4E). No apparent differences in T cell numbers or function were also reflected in identical levels of persisting viral mRNA in both brains and spinal cords (Figure 4F). These data indicated that viral persistence was not regulated by Foxp3^+^ Tregs, distinct from CLN. Finally, no overt differences in demyelination in Foxp3^+^ Treg ablated mice indicated a minor, if any, role of this Treg subset in CNS tissue damage (Figure 4G). These data demonstrated that Foxp3^+^ Tregs control T cell activation and inflammatory responses within CLN but have no effect on CNS inflammation and pathology during chronic JHMV infection.

### Foxp3^+^ Tregs Do Not Regulate Virus-Specific or SR CD4^+^ T Cells in the CNS

**Figure 5:**
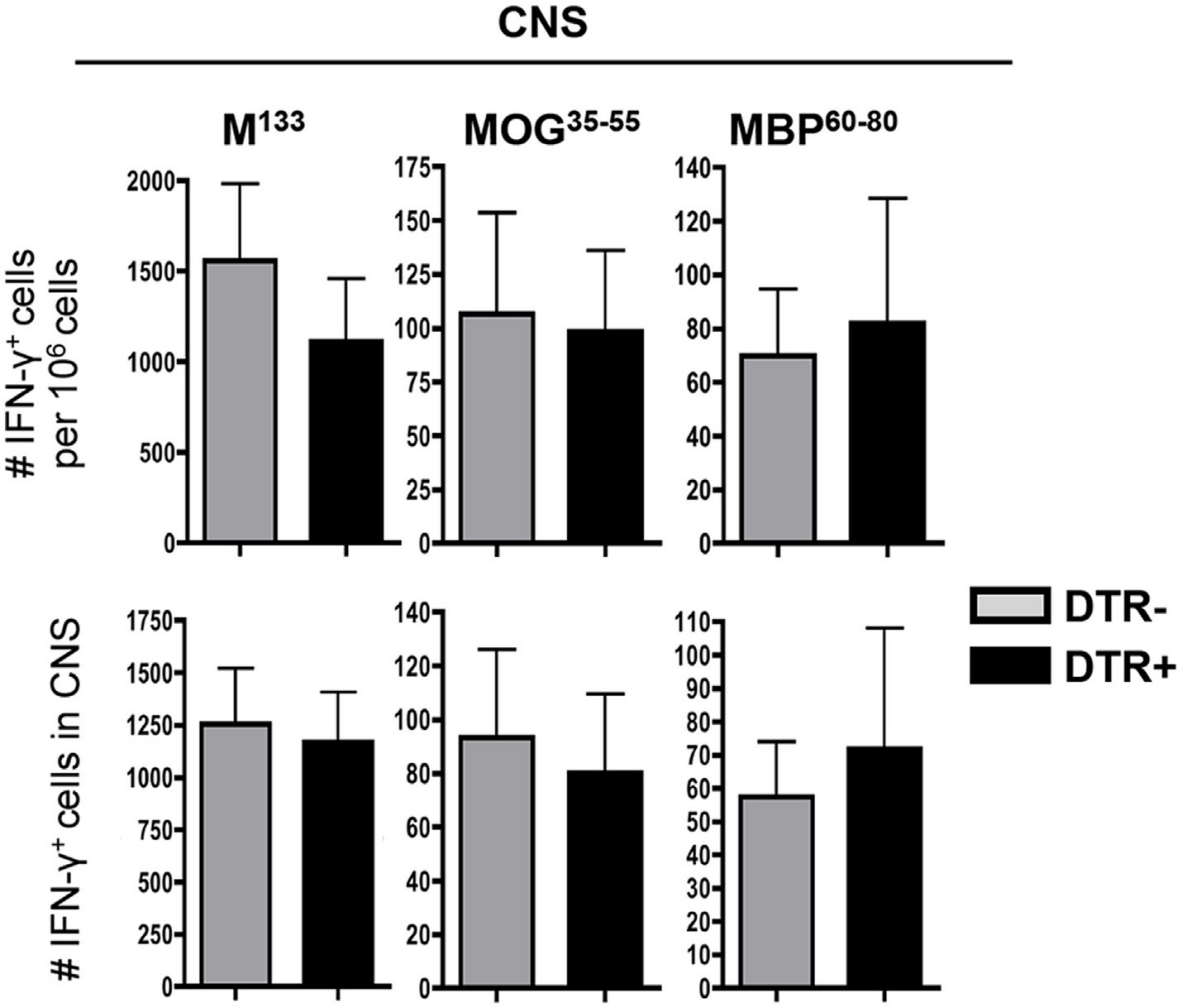
**Foxp3^+^ Tregs do not affect CNS SR T cells during chronic JHMV infection**. Frequencies and absolute numbers of virus-specific M^133^, myelin-specific MOG^35−55^, and MBP^60−80^ CD4^+^ T cells in CNS of JHMV-infected DTR^−^ and DTR^+^ mice treated with DT between d21 and d28 p.i. determined by ELISPOT. A number of spots detected in wells without stimulation were subtracted to exclude non-specific CD4^+^ T cells. Data represent the mean of two separate experiments with seven to eight pooled mice per group per experiment.

While neither CNS antiviral function nor demyelination were affected by Foxp3^+^ Treg depletion, a potential increase in virus-specific and SR CD4^+^ T cells may be unapparent due to their low frequencies in the CNS. Specifically, SR CD4^+^ T cell frequencies are low within the CNS, although higher than in CLN (13). To verify that their increased numbers in CLN do not lead to enhanced circulation and recruitment to the CNS, we used ELISPOT to determine frequencies of both virus-specific and SR T cells in the CNS. Frequencies as well as total numbers of IFN-γ secreting M^133^-specific CD4^+^ T cells were indeed similar in the CNS of both groups (Figure 5). Contrasting the CLN, both frequencies and total numbers of IFN-γ secreting MOG^35−55^- and MBP^60−80^-specific CD4^+^ T cells were also identical within the CNS of DT-treated DTR^−^ and DTR^+^ mice (Figure 5). These data were consistent with similar demyelination and supported a limited role for Foxp3^+^ Tregs in controlling the CNS SR CD4^+^ T cell response during chronic JHMV infection.

**Figure 6:**
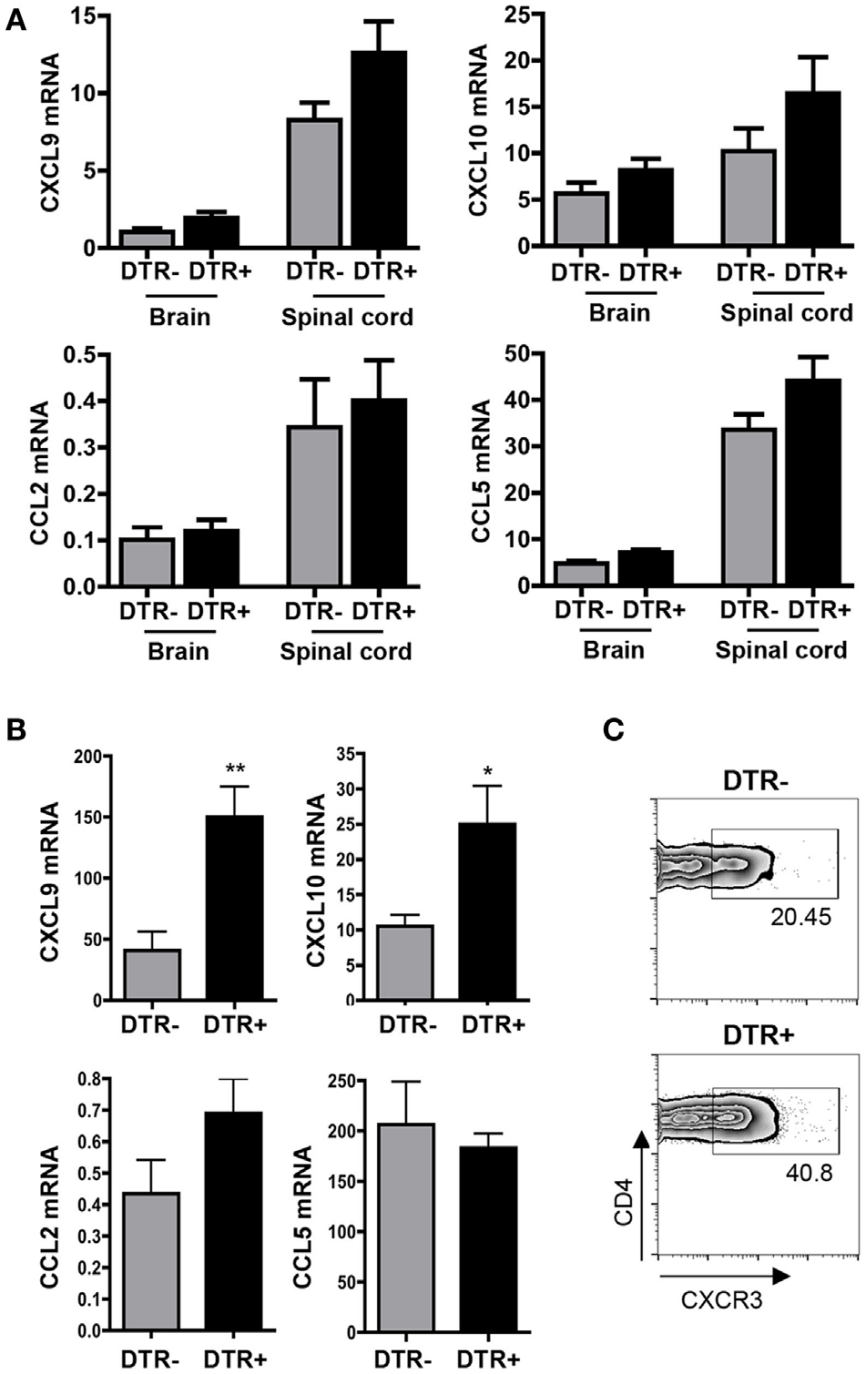
**Increased chemokine expression within CLN of Foxp3^+^ Treg depleted mice**. mRNA expression of CXCL9, CXCL10, CCL2, and CCL5 relative to GAPDH at d28 p.i., 1 week after DT treatment, in brain, and spinal cord **(A)**, and CLN **(B)** of DTR^−^ and DTR^+^ mice with *n* = 5–6 individual mice per group. *p < 0.05, **p < 0.01, Mann–Whitney test. **(C)** Representative flow cytometry density plots depicting CXCR3 surface expression within CLN-derived CD4^+^ T cells in DT-treated DTR^−^ and DTR^+^ mice at d28 p.i. Data are representative of two separate experiments.

Increased T cell numbers and activation within CLN after Foxp3^+^ Treg depletion, but no differences in the CNS, implicated impaired T cell trafficking. Following JHMV infection, T cell migration to the CNS is regulated by CXCR3, as well as CCR2 and CCR5 (33–35). However, expression of their respective ligands, CXCL9, CXCL10, CCL2, and CCL5 was not affected by Foxp3^+^ Treg ablation within brain or spinal cord (Figure 6A). However, Foxp3^+^ Tregs have been reported to limit inflammatory chemokine expression within draining lymph nodes, thereby promoting T cell egress (36). Elevated T cell activation and IFN-γ mRNA in CLN were indeed associated with significantly increased levels of IFN-γ inducible CXCL9 and CXCL10 mRNA levels in CLN of Foxp3^+^ Treg depleted DTR^+^ mice (Figure 6B). Similarly, CXCR3 expression on CD4^+^ T cells was upregulated in CLN (Figure 6C). By contrast, there were no differences in CCL2 and CCL5 mRNA expression (Figure 6B), indicating a preferential increase of IFN-γ regulated chemokines (37, 38). These results supported the notion that enhanced and/or prolonged interaction of CXCR3 ligand expressing APC with CXCR3^+^ T cells may promote retention, thereby limiting T cell egress into circulation (39). Overall, these results indicate that Foxp3^+^ Tregs mediate suppressive function primarily within CLN and are more potent at controlling peripheral SR T cell activation than virus-specific T cells during chronic JHMV infection.

### Reduced Tr1 Cell Response Correlates with Increased CNS SR T Cells

**Figure 7:**
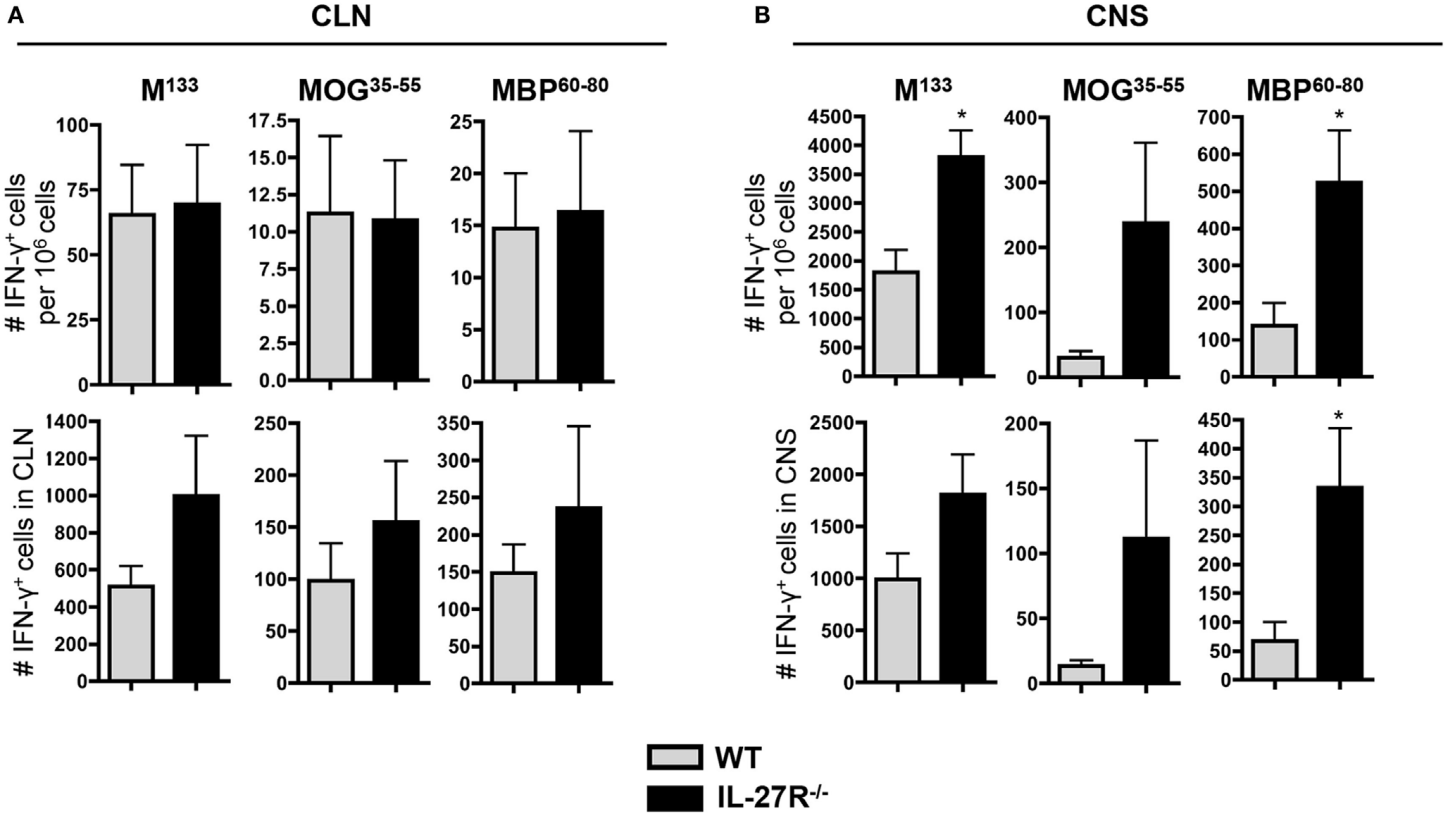
**Increased CNS SR T cell response in absence of Tr1 cells**. Frequencies and absolute numbers of virus-specific M^133^, MOG^35−55^, and MBP^60−80^ specific CD4^+^ T cells in CLN **(A)** and CNS **(B)** derived cells from JHMV-infected WT and IL-27R^−/−^ mice at d30 p.i. determined by ELISPOT. A number of spots detected in wells without stimulation were subtracted to exclude non-specific CD4^+^ T cells. Data represent the mean of two separate experiments with seven to eight pooled mice per group per experiment. *p < 0.05, Mann–Whitney test.

Unaltered SR T cell responses within the CNS of DT-treated DTR^+^ mice indicated a Foxp3-independent component regulating the CNS autoimmune response during chronic JHMV infection. In addition to Foxp3^+^ Tregs, IL-10-producing Tr1 cells also regulate autoimmune responses, including during MS (40, 41). Tr1 cells are drastically reduced in JHMV-infected mice deficient in IL-27 signaling, a cytokine promoting Tr1 cell response (15). Analysis of IL-27R^−/−^ mice further demonstrated that IL-27 does not play a pro-inflammatory role or significantly alter CD8 T cell function during JHMV infection but is rather a major regulator of the Tr1 response (15). Myelin-specific CD4^+^ T cell responses were therefore compared in infected WT and IL-27R^−/−^ mice at d30 p.i., the peak of the SR T cell response (13). Opposing the results in Foxp3^+^ Treg depleted mice, frequencies and numbers of both virus- and myelin-specific CD4^+^ T cells were similar within CLN of IL-27R^−/−^ mice compared to controls (Figure 7A). Furthermore, frequencies of both virus-specific and SR CD4^+^ T cells were significantly increased within the CNS of IL-27R^−/−^ mice compared to WT mice (Figure 7B). Interestingly, alteration of CD4^+^ T cell frequencies in the absence of Tr1 cells was more prominent for myelin-specific compared to virus-specific T cells (fivefold versus twofold, respectively), indicating preferential action on SR T cells. Moreover, despite similar overall inflammatory cell numbers, numbers of virus and SR CD4^+^ T cells were increased in the absence of Tr1 cells (Figure 7B). Tr1 cells thus do not appear to regulate peripheral SR CD4^+^ T cell responses but act preferentially within the CNS during chronic JHMV infection. These data indicate that regulation of SR CD4^+^ T cell response during chronic JHMV infection is dependent upon Tr1 cells within the CNS, contrasting the specific role of Foxp3^+^ Tregs within CLN.

## Discussion

Viral infections have long been associated with the induction and reoccurrence of autoimmune attacks, including MS (1). Nevertheless, a firm association between virus and MS remains elusive, as active viral replication has thus far never been detected within CNS tissue of MS patients. On the other side, a protective role of viral infections against autoimmune diseases has also been proposed (8), but the mechanisms preventing autoimmunity after viral infections remain largely unknown. Among MS murine models, JHMV infection reproduces several aspects of MS pathogenesis (12). Importantly, tissue damage occurs temporally remote from viral infection as indicated by peak demyelination after elimination of infectious virus (12). Moreover, lesion size is constrained by IL-10 during persistence independent of viral load (42). Lastly, myelin loss correlates with the peripheral activation of myelin-specific CD4^+^ T cells, which access the CNS but decline with time despite ongoing demyelination (13). The apparent control of the endogenous SR T cells suggested suppressive mechanisms prevent ascending clinical autoimmune disease during persistent infection. JHMV infection thus represents a unique model to assess how viral infection may prevent autoimmunity.

As Foxp3^+^ Tregs are among the regulatory mechanisms dampening JHMV pathogenesis, their implication in controlling SR CD4^+^ T cells was of particular interest. The data herein are novel in demonstrating that Foxp3^+^ Treg abrogation during persistence preferentially enhanced endogenously primed, peripheral SR CD4^+^ T cells but had no effect on CNS localized SR T cells or tissue damage during chronic JHMV infection. These observations are consistent with a previous report by Trandem et al. (20) showing that naive Foxp3^+^ Tregs adoptively transferred into acutely JHMV-infected mice were not detected within the CNS but rather exerted prominent function within CLN by dampening dendritic cell activation, T cell proliferation, and pro-inflammatory cytokine expression. Foxp3^+^ Treg dependent downregulation of CLN inflammatory response thus correlated with limited CNS inflammation and demyelination without affecting viral persistence (20). Our results on regulation of SR T cells during persistence also corroborated control of transferred SR CD4^+^ T cell proliferation by Foxp3^+^ Tregs during a heterologous MHV infection (21). A primary role of Foxp3^+^ Tregs within draining lymph nodes was also observed in other infectious models, as well as during cancer and autoimmunity (36, 43–45). Nevertheless, our data differ from results by Trandem et al. with respect to the direct correlation between decreased peripheral inflammation by Foxp3^+^ Tregs with decreased CNS cellular infiltration and demyelination. Despite extensive expansion of the inflammatory response within the CLN of mice depleted of Foxp3^+^ Tregs during persistence, no alteration of CNS inflammation or tissue damage was evident. These differences may reside in the differential properties of the blood–brain barrier (BBB) between acute and chronic JHMV infection, as BBB permeability is restored to naive levels after day 14 p.i. (46), thus potentially limiting effector T cell access into the CNS after Foxp3^+^ Treg depletion. Further, enlarged CLN associated with increased chemokine expression indicated restricted T cell egress is responsible for the lack of altered CNS inflammation. Retention of effector T cells within the draining lymph nodes following Foxp3^+^ Treg depletion was also observed during herpes simplex viral (HSV) infection. In this model, Treg-mediated suppression of chemokine production within lymph nodes facilitated effector T cell egress and trafficking to the infection site (36). While a similar mode of Treg action is supported by our studies, Treg supplementation, rather than depletion, enhanced chemokine production and T effector cell retention in lymph nodes during experimental autoimmune encephalomyelitis (EAE), thereby diminishing CNS T cell migration (47). All together, these diverse CNS inflammation models suggest that Foxp3^+^ Treg can modulate effector T cells in draining lymph nodes both at the expansion phase as well as egress phase. The relative balance between expansion and local cytokine/chemokine expression then ultimately determines egress into circulation and access to the CNS.

Irrespective of the overall common suppressive action in draining LN during viral infection, the function of endogenous Foxp3^+^ Treg migrating to and residing in the CNS throughout persistence remains obscure. Despite the prolonged reduction of Tregs by DT-mediated depletion, we found no evidence of altered pathogenesis. Regulation of the CNS autoimmune responses by Foxp3^+^ Tregs is also ineffective during EAE (48). Despite Foxp3^+^ Treg accumulation within the inflamed CNS, their functions were abrogated by pro-inflammatory cytokines IL-6 and TNF. Expression of both cytokines during JHMV infection (49) suggests they may have similar suppressive activity on local Foxp3^+^ Treg. Alternatively, a potential deficit of Foxp3^+^ Treg access to CNS demyelinating lesions, with perivascular retention, may explain their inability to regulate CNS inflammation and will require further investigation. It is also possible that residual Foxp3^+^ Tregs detected 1 week after DT treatment may be sufficient to control CNS inflammation. The minor Foxp3^+^ Treg population detected at day 28 p.i. presumably comprises rebound Tregs, as well as a small proportion of DT insensitive Foxp3^+^ cells detected in adult DEREG mice (23). While rebound Tregs are dysfunctional (28), DT-resistant Foxp3^+^ Tregs have been shown to protect against lethal autoimmunity and may thus suffice to control the SR CD4^+^ T cells during chronic JHMV infection (23). It nevertheless remains intriguing that residual Foxp3^+^ Tregs in CLN appear to exert more potent suppressive capacities than in the CNS.

In contrast to the lack of Foxp3^+^ Treg suppressive function within the CNS, IL-27R^−/−^ mice displayed increased virus and SR CD4^+^ T cell responses, implicating Tr1 cells as a major regulator of CNS inflammation during chronic JHMV infection. Impaired IL-27 signaling was associated with decreased IL-10^+^ virus-specific CD4^+^ T cells and delayed viral control (15). The present results indeed confirmed that Tr1 cells dampened the virus-specific Th1 cell response within the CNS. However, a greater increase of SR CD4^+^ T cells was observed in the CNS of IL-27R^−/−^ mice, indicating that Tr1 cells are also critical in limiting autoimmunity during chronic JHMV infection. It remains to be determined whether Tr1 cells restricting SR CD4^+^ T cell response during chronic JHMV infection are myelin-specific, as Tr1 regulatory function efficacy was shown to be dependent upon antigen specificity (50, 51). The mechanisms associated with Tr1-dependent suppression of CNS SR T cells may involve IL-10 secretion, granzyme B, and perforin production, inhibitory molecule expression, or metabolic regulation (52). Finally, the implication of SR CD4^+^ T cells in promoting tissue damage and clinical autoimmunity during JHMV infection remain unclear, as increased myelin-specific CD4^+^ T cells in IL-27R^−/−^ mice correlated with a moderate decrease, rather than expected increase in demyelination (15). It cannot be excluded that despite their increased frequency, the overall low numbers of myelin-specific CD4^+^ T cells are insufficient to achieve effector threshold levels required to affect myelin loss or tissue damage. Prolonged studies may be necessary to reveal altered tissue damage, as demyelination in IL-27R^−/−^ mice was quantified only until day 30 p.i. (15). It also remains a possibility that compensatory mechanisms occur in the absence of Tr1 cells. IL-10 is critical in limiting demyelination during JHMV persistence and is produced by both Foxp3^+^ Tregs and Tr1 cells. Surprisingly however, no difference in IL-10 mRNA levels was detected between WT and IL-27R^−/−^ mice during chronic JHMV infection (42), indicating another cell type may compensate for the lack of Tr1 cells.

Altogether, our study demonstrates the importance of distinct Treg populations in controlling SR T cells both at the induction and effector site during chronic encephalomyelitis. These are the first data to show a differential role of Foxp3^+^ Tregs versus Tr1 cells in limiting SR CD4^+^ T cells in CLN and CNS, respectively, thereby maximizing prevention of autoimmunity. Deciphering the mechanisms by which distinct Treg subsets function in specific anatomical locations and different inflammatory conditions may lead to potential future therapeutic strategies to prevent or limit autoimmunity following viral infections.

## Author Contributions

CS designed and performed experiments, collected and interpreted the data, and wrote the manuscript. DH performed and analyzed histopathological experiments. CB and SS designed the research, provided materials, interpreted the data, and wrote the manuscript. All authors approved the final manuscript.

## Conflict of Interest Statement

The authors declare that the research was conducted in the absence of any commercial or financial relationships that could be construed as a potential conflict of interest.

## Abbreviations

APC, antigen-presenting cell; BBB, blood–brain barrier; CLN, cervical lymph node; CNS, central nervous system; DT, diphtheria toxin; DTR, diphtheria toxin receptor; EAE, experimental autoimmune encephalomyelitis; HSV, herpes simplex virus; LFB, Luxol fast blue; MHV, mouse hepatitis virus; MBP, myelin basic protein; MOG, myelin oligodendrocyte glycoprotein; MS, multiple sclerosis; SR, self-reactive; TMEV, Theiler’s murine encephalomyelitis virus; Tregs, regulatory T cells; Tr1, type 1 regulatory cells.
